# Effects of PCSK9 inhibitors on HDL cholesterol efflux and serum cholesterol loading capacity in familial hypercholesterolemia subjects: a multi-lipid-center real-world evaluation

**DOI:** 10.3389/fmolb.2022.925587

**Published:** 2022-07-19

**Authors:** Marcella Palumbo, Antonina Giammanco, Francesco Purrello, Chiara Pavanello, Giuliana Mombelli, Antonino Di Pino, Salvatore Piro, Angelo Baldassare Cefalù, Laura Calabresi, Maurizio Averna, Franco Bernini, Francesca Zimetti, Maria Pia Adorni, Roberto Scicali

**Affiliations:** ^1^ Department of Food and Drug, University of Parma, Parma, Italy; ^2^ Department of Health Promotion, Mother and Child Care, Internal Medicine and Medical Specialties (ProMISE)—University of Palermo, Palermo, Italy; ^3^ Department of Clinical and Experimental Medicine, University of Catania, Catania, Italy; ^4^ Centro E. Grossi Paoletti, Dipartimento di Scienze Farmacologiche e Biomolecolari, Università degli Studi di Milano, Milano, Italy; ^5^ Centro Dislipidemie, ASST Grande Ospedale Metropolitano Niguarda, Milano, Italy; ^6^ Department of Medicine and Surgery, Unit of Neuroscience, University of Parma, Parma, Italy

**Keywords:** PCSK9 inhibitors, cholesterol efflux capacity, cholesterol loading capacity, familial hypercholesterolemia, cardiovascular risk

## Abstract

Proprotein convertase subtilisin/kexin type 9 (PCSK9), beyond regulating LDL cholesterol (LDL-c) plasma levels, exerts several pleiotropic effects by modulating lipid metabolism in extrahepatic cells such as macrophages. Macrophage cholesterol homeostasis depends on serum lipoprotein functions, including the HDL capacity to promote cell cholesterol efflux (CEC) and the serum capacity to promote cell cholesterol loading (CLC). The aim of this observational study was to investigate the effect of PCSK9 inhibitors (PCSK9-i) treatment on HDL-CEC and serum CLC in patients with familial hypercholesterolemia (FH). 31 genetically confirmed FH patients were recruited. Blood was collected and serum isolated at baseline and after 6 months of PCSK9-i treatment. HDL-CEC was evaluated through the main pathways with a radioisotopic cell-based assay. Serum CLC was assessed fluorimetrically in human THP-1 monocyte-derived macrophages. After treatment with PCSK9-i, total cholesterol and LDL-c significantly decreased (−41.6%, *p* < 0.0001 and −56.7%, *p* < 0.0001, respectively). Total HDL-CEC was not different between patients before and after treatment. Conversely, despite no changes in HDL-c levels between the groups, ABCG1 HDL-CEC significantly increased after treatment (+22.2%, *p* < 0.0001) as well as HDL-CEC by aqueous diffusion (+7.8%, *p* = 0.0008). Only a trend towards reduction of ABCA1 HDL-CEC was observed after treatment. PCSK9-i significantly decreased serum CLC (−6.6%, *p* = 0.0272). This effect was only partly related to the reduction of LDL-c levels. In conclusion, PCSK9-i treatment significantly increased HDL-CEC through ABCG1 and aqueous diffusion pathways and reduced the serum CLC in FH patients. The favorable effect of PCSK9-i on functional lipid profile could contribute to the cardiovascular benefit of these drugs in FH patients.

## 1 Introduction

Beyond the main effect of the proprotein convertase subtilisin/kexin type 9 (PCSK9) on low density lipoprotein cholesterol (LDL-c) plasma levels, several extrahepatic properties affecting cholesterol metabolism have been described (Yurtseven et al., 2020). Among these, it has been demonstrated that PCSK9 interferes with the cholesterol efflux from macrophages, the first step of the atheroprotective process of reverse cholesterol transport. Macrophage cholesterol efflux is promoted by the aqueous diffusion (AD) but primarily by the activity of membrane transporters such as ATP binding cassette A1 (ABCA1) and ATP binding cassette G1 (ABCG1) (Pownall et al., 2021); in this pathways, high density lipoprotein (HDL) is the principal cholesterol efflux extracellular acceptor and its subclasses have different affinity to the single cholesterol transporters (Adorni et al., 2021). In macrophages PCSK9 directly inhibits the ABCA1-mediated cholesterol efflux through a down-regulation of the ABCA1 gene and ABCA1 protein expression (Adorni et al., 2017) and it enhances the macrophage cholesterol content by increasing the expression of the scavenger receptors involved in uncontrolled cholesterol internalization (Badimon et al., 2021). Since these effects may influence the pathogenesis of atherosclerosis by promoting foam cell formation, these cells are the *primum movens* of atherosclerotic injury (Chistiakov et al., 2017). In this context, it could be interesting to investigate novel possible anti-atherosclerotic properties of PCSK9 inhibition independently of the reduction of LDL-c levels. Macrophage cholesterol content is also highly influenced by serum lipoprotein functions, i.e., the HDL capacity to promote cholesterol efflux (CEC) (Adorni et al., 2021) and the serum capacity to load macrophages with cholesterol (CLC) (Adorni et al., 2012). It was previously shown that HDL-CEC predicted future cardiovascular (CV) events in the general population (Rohatgi et al., 2014; Ritsch et al., 2015; Saleheen et al., 2015; Khera et al., 2017) and in patients with acute coronary syndrome (ACS); in particular, in the PREDIMED study higher levels of CEC were associated with a lower risk of ACS independently of other risk factors such as HDL (Soria-Florido et al., 2020). In this context, HDL-CEC could be a useful cardiovascular biomarker in high cardiovascular risk subjects such as familial hypercholesterolemia (FH) patients (Cuchel et al., 2018).

Since lipid trafficking depends on cholesterol efflux and influx—the latter promoting the cholesterol storage in macrophages (Weibel et al., 2014)—CLC represents another functional index of circulating lipoprotein-related serum atherogenicity and it is raised in pathological lipid homeostasis conditions leading to atherosclerotic cardiovascular disease (ASCVD) (Adorni et al., 2012). Previous studies reported that these serum functional lipid-related parameters could be modulated by pharmacological treatments (Ronda et al., 2015; Greco et al., 2020; van Velzen et al., 2021) with a potential cardiovascular benefit.

PCSK9 inhibitors (PCSK9-i) act reducing LDL-c and previous trials showed that they are able to reduce ASCVD (Murphy et al., 2019; Steffens et al., 2020); thus, PCSK9-i treatment is currently a useful lipid-lowering option in FH subjects (Stein et al., 2012). As concerns the PCSK9 monoclonal antibodies evolocumab and alirocumab, while several data have been reported on their LDL-c lowering effect with only a modest increasing effect on HDL-c levels (Sabatine et al., 2017), few data exist on their impact on HDL-CEC and even no data are present on serum CLC. Thus, we aimed to study the effects of PCSK9 monoclonal antibody treatment on HDL-CEC and its pathways in a cohort of heterozygous FH patients. Moreover, we evaluated the effect of PCSK9-i treatment on cholesterol loading in macrophages by assessing serum CLC.

## 2 Methods

### 2.1 Study design and population

It was an open-label, observational study concerning previously genetically confirmed FH subjects (Averna et al., 2017). All participants were enrolled from the Lipid Centers of the “Azienda Ospedaliera Universitaria Policlinico” of Palermo, Sicily, Italy; Niguarda Hospital of Milano, Italy; and Garibaldi Hospital/University of Catania, Sicily, Italy, from January 2018 to September 2020. For all participants, at the time of enrollment the ages were 18–70 years and LDL-c was beyond the recommended targets (Mach et al., 2020) despite high-intensity statins (atorvastatin 40–80 mg, rosuvastatin 20–40 mg) plus ezetimibe for at least 6 months. After a 12 h fast, hematological and clinical evaluations were performed on all participants; furthermore, biochemical analyses were obtained at baseline (T0) and after 6 months (T1) of PCSK9-i treatment. The baseline evaluations of body mass index, arterial hypertension as well as smoking and glycemic status were performed in all participants; moreover, detailed pharmacological intake as well as ASCVD history were obtained. LDL-c target was defined according to 2019 European Society of Cardiology (ESC)/European Atherosclerosis Society (EAS) guidelines for the management of dyslipidemias (Mach et al., 2020). Subjects taking non-statin lipid-lowering therapy were excluded from the study. All procedures were followed in accordance with the ethical standards of the local institutional committees on human experimentation and according to the Helsinki Declaration of 1964, as revised in 2013.

### 2.2 HDL cholesterol efflux capacity (CEC)

The capacity of HDLs to promote cholesterol efflux (HDL-CEC) was evaluated on patients’ serum HDL fraction. HDL fraction was isolated from the apoB-containing lipoproteins by treating whole serum with polyethylene glycol (Asztalos et al., 2005). This technique, which leads to obtain biological samples containing only HDL, is comparable to the isolation of HDL by ultracentrifugation for the study of cholesterol efflux capacity (Horiuchi et al., 2019).

Sera were slowly defrosted in ice before this procedure to impede lipoprotein remodeling. HDL-CEC, through the main pathways, was evaluated by a standardized radioisotopic cell-based technique (van Velzen et al., 2021).

#### 2.2.1 Total, aqueous diffusion and ATP-binding cassette transporter A1 HDL-CEC

Total HDL-CEC, the pathway inversely related to cardiovascular risk, and its main contributions, the aqueous diffusion (AD) and the cholesterol efflux mediated by the ATP-binding cassette transporter A1 (ABCA1), were assessed in a murine macrophage cell model (J774). In particular, to evaluate AD were used J774 in basal conditions, whereas, for total HDL-CEC J774 cells were treated with a cAMP analogue (cpt-cAMP 0.3 mM; Sigma Aldrich, Milano, Italy) to induce ABCA1 expression (Favari et al., 2005). The specific contribution of ABCA1 was calculated as the difference between total HDL-CEC and AD HDL-CEC. J774 were seeded in 10% fetal calf serum (FCS) containing Dulbecco’s Modified Eagle Medium (DMEM) (both FCS and DMEM from Euroclone, Milano, Italy) in the presence of antibiotics (penicillin–streptomycin from Thermo Fisher Scientific, MA, United States). After 24 h, J774 cells were radiolabelled for 24 h with (1,2-^3^H) cholesterol (PerkinElmer, Waltham, MA, United States) at 2 μCi/ml in the presence of 2 μg/ml of an inhibitor of the cholesterol esterifying enzyme acyl-coenzyme A: cholesterol acyltransferase (ACAT) (Sandoz 58035; Sigma-Aldrich) to prevent intracellular cholesteryl esters formation. Cells were incubated in 0.2% bovine serum albumin (BSA) containing DMEM, in presence or absence of cAMP analogue, for 18 h (BSA from Sigma-Aldrich, Milano, Italy). Cells were then incubated with the HDL sera fraction from FH patients before and after 6 months of PCSK9-i treatment at 2% (v/v) in medium for 4 h. HDL-CEC was expressed as the percentage of ^3^H-cholesterol released in the medium over the total cell radioactivity. To evaluate proper cell responsiveness, internal positive controls including lipid-free human apolipoprotein A-I (Sigma-Aldrich, Milano, Italy) and a standard serum HDL fraction isolated from a pool of normolipidemic subjects, were tested with serum samples in each assay. The HDL-CEC values of control samples were used to minimize the inter-assay variability. Intra-assay variation coefficients for HDL-CEC assays were <10%.

#### 2.2.2 ATP binding cassette transporter G1 HDL-CEC

HDL-CEC mediated by the ATP binding cassette transporter G1 (ABCG1) was evaluated in Chinese hamster ovary (CHO) cells transfected and not transfected with the human ABCG1 gene. The specific ABCG1 contribution was calculated as the difference between HDL-CEC evaluated in ABCG1-transfected CHO cells and HDL-CEC measured in non-transfected cells. Specifically, CHO cells were plated in 10% FCS containing Ham’s F-12 (FCS from Euroclone, Milano, Italy, and Ham’s F-12 from Lonza, Durham, NC, United States) in presence of antibiotics (penicillin–streptomycin and Zeocin from Thermo Fisher Scientific, Waltham, MA, United States). CHO cells, after 24 h of labelling with [1,2-^3^H] cholesterol at 1 μCi/ml, were equilibrated in 0.2% BSA-containing medium for 90 min. Cells were successively incubated for 6 h with the HDL sera fraction from FH patients before and after 6 months of PCSK9-i treatment at 1% (v/v) in medium. HDL-CEC was expressed as the percentage of ^3^H-cholesterol released in the medium over the total cell radioactivity. To evaluate proper cell responsiveness, internal positive controls including human isolated HDLs and the HDL fraction of a standard serum isolated from a pool of normolipidemic subjects were tested with the serum samples in each assay. Human standard HDLs (d 1.063–1.21 g/ml) were purified by serial ultracentrifugation from the plasma of healthy volunteers. The HDL-CEC values of control samples were used to minimize the inter-assay variability, which was <10%.

### 2.3 Serum cholesterol loading capacity (CLC)

Also for this assay, patient’s sera were slowly defrosted in ice immediately before analysis, to avoid lipoprotein remodeling (Adorni et al., 2019a). The capacity of serum to promote cholesterol loading (CLC) was analyzed in human monocyte-derived THP-1 macrophages using a fluorometric assay (Zimetti et al., 2006). Human THP-1 monocytes were grown in 10% FCS containing Roswell Park Memorial Institute (RPMI) 1640 (both from Euroclone, Milano, Italy) in the presence of antibiotics (penicillin–streptomycin from Thermo Fisher Scientific, Waltham, MA, United States). To allow differentiation into macrophages, cells were seeded in the presence of 100 ng/ml phorbol 12-myristate 13-acetate (PMA) (Sigma-Aldrich, Milano, Italy) for 72 h. Cells were then exposed to 5% human lipoprotein-deficient serum (Sigma-Aldrich, Milano, Italy) for 24 h and subsequently incubated with 10% (v/v) whole serum from FH patients before and after 6 months of PCSK9-i treatment for 24 h. At the end of treatment, cell monolayers were lysed by 1% sodium cholate solution (Sigma-Aldrich, Milano, Italy), supplemented with 10 U/ml DNase (Sigma-Aldrich, Milano, Italy). Intracellular cholesterol was then measured fluorometrically using the Amplex Red Cholesterol Assay Kit (Molecular Probes, Eugene, OR, United States) following the manufacturer’s instructions. To measure cell protein content, an aliquot of cell lysate was analyzed with the bicinchoninic acid assay (Thermo Fisher Scientific, Waltham, MA, United States). Serum CLC was expressed as micrograms of cholesterol per milligram of protein. To verify proper cell responsiveness, internal positive controls including sera from a pool of hypercholesterolemic and normolipidemic subjects were analyzed with serum samples in each experiment. The relative CLC values of control samples were used to minimize the inter-assay variability. Intra-assay CV for the CLC assays was <10%.

### 2.4 Statistical analysis

G*Power software (Düsseldorf, Germany) was used for *a priori* sample size calculation. Based on data from a previous study (Ogura et al., 2016), a mean of total HDL-CEC of 0.80 ± 0.14 was assumed in FH patients with ASCVD, versus a mean of 0.92 ± 0.11 in FH patients without significant ASCVD. With an alpha of 0.05 and power of 80%, a sample size of at least 30 FH subjects with or without ASCVD was required.

Statistical analyses were carried out using GraphPad Prism version 7.0 (GraphPad Software, La Jolla, CA, United States). Each sample was analyzed in triplicate. Data are reported as mean ± SD for parameters presenting a normal distribution, or as median with interquartile range (IQR) (25th to 75th percentile), for parameters with skewed distribution. The D'Agostino & Pearson normality test was performed to assess normality of distribution. The paired two-tailed Student’s t-test, for parameters with normal distribution, and the Wilcoxon matched-pairs signed rank test, for parameters with skewed distribution, were utilized to evaluate differences between FH patients before and after 6 months of PCSK9-i treatment.

Correlation analyses, using a univariate logistic regression, were performed to evaluate the relationship between parameters. Pearson or Spearman correlation coefficients (r) were stated for data with normal and skewed distribution, respectively. Statistical significance was defined as *p* < 0.05.

## 3 Results

### 3.1 Patients’ characteristics

After the inclusion criteria evaluation, a total of 31 FH subjects participated at the study. In agreement with the 2019 ESC/EAS guidelines for the management of dyslipidemias and the national PCSK9-i prescriptive regulation (Mach et al., 2020; Pasta et al., 2020), PCSK9-i treatment was started in all FH subjects; specifically, six subjects added alirocumab 150 mg and 25 subjects added evolocumab 140 mg, every 2 weeks.

Table 1 shows the characteristics of the study population. The population was homogeneous in terms of sex, age and BMI, while more than half of patients had a previous CV event. In particular, the majority of patients suffered from coronary artery disease while cerebrovascular disease was present in 11.8% of population; finally, 17.6% of patients were affected by peripheral artery disease. All FH patients had heterozygous LDLR genetic variants and the most prevalent mutation class was amino acid change. Finally, the majority of FH patients were on rosuvastatin 20 mg, while the percentage of patients on atorvastatin 40 mg was 41.9% as well as the prevalence of assumed antihypertensive treatment.

**TABLE 1:** Characteristics of the study population.

Demographic Characteristics	
N	31
Age — years ±SD	57 ± 11
Male — *n*. (%)	16 (51.6)
BMI — Kg/m^2^	25 ± 3
Smoking — *n*. (%)	6 (19.4)
Diabetes Mellitus — *n*. (%)	1 (3.8)
ASCVD — *n*. (%)	17 (54.8)
- Coronary artery disease, *n* (%)	12 (70.6)
- Cerebrovascular disease, *n* (%)	2 (11.8)
- Peripheral artery disease, *n* (%)	3 (17.6)
Hypertension — *n*. (%)	13 (41.9)
Mutation class	
Amino acid change, *n* (%)	17 (54.8)
Null allele, *n* (%)	14 (45.2)
FH phenotype	
Heterozygous FH, *n* (%)	31 (100.0)
Treatment	
Antihypertensive therapy, *n* (%)	13 (41.9)
Antiplatelet therapy, *n* (%)	17 (54.8)
High-intensity statin therapy	
Atorvastatin 40 mg, *n* (%)	13 (41.9)
Rosuvastatin 20 mg, *n* (%)	18 (58.1)
Ezetimibe, *n* (%)	31 (100.0)

BMI, body mass index; ASCVD, atherosclerotic cardiovascular disease; FH, familial hypercholesterolemia; LDLR, low-density lipoprotein receptor; PCSK9-i, proprotein convertase subtilisin/kexin type 9 inhibitors; SD, standard deviation. Data are presented as mean ± SD, percentages, or median (interquartile range, IQR).

As concerns the lipid profile, we observed a significative reduction of total cholesterol (−41.6%, *p* < 0.0001), and LDL-c (−56.7%, *p* < 0.0001) after 6 months of PCSK9-i treatment; specifically, more than half of patients achieved the recommended LDL-c target according to the recent guidelines (Mach et al., 2020).

No changes in HDL-c levels were observed (*p* = 0.4965). A trend to a reduction in triglycerides levels occurred, however, without reaching statistical significance (*p* = 0.0706) (Table 2). In two secondary analyses, after stratification of FH patients according to smoking and hypertension respectively, similar results were obtained after 6 months of PCSK9-i treatment (Supplementary Tables S1, S2, Supplementary Material).

**TABLE 2:** Lipid profile characteristics of study population at baseline and after 6 months of PCSK9-i treatment.

Lipid profile— mg/dl	Before treatment HeFH (*n* = 31)	After 6-month PCSK9-i treatment HeFH (*n* = 31)	*p*-value
TC	238 (205–287)	139 (113–181)	**<0.0001**
LDL-c	140 (109–188)	61 (42–110)	**<0.0001**
- *LDL-c target, n (%)*	-	*16 (51.6)*	-
HDL-c	53 ± 11	53 ± 9	0.4965
TG	112 (74–130)	95 (72–118)	0.0706

HDL-c, High Density Lipoproteins; HeFH, heterozygous familial hypercholesterolemia; LDL-c, Low Density Lipoproteins; PCSK9-i, proprotein convertase subtilisin/kexin type 9 inhibitors; TC, total cholesterol; TG, triglyceride. Data are presented as mean ± SD, or median (interquartile range, IQR) according to normal or skewed distribution respectively. Values in bold indicate statistically significant results.

No cardiovascular events occurred during 6 months of PCSK9-i treatment.

### 3.2 HDL cholesterol efflux capacity

**FIGURE 1:**
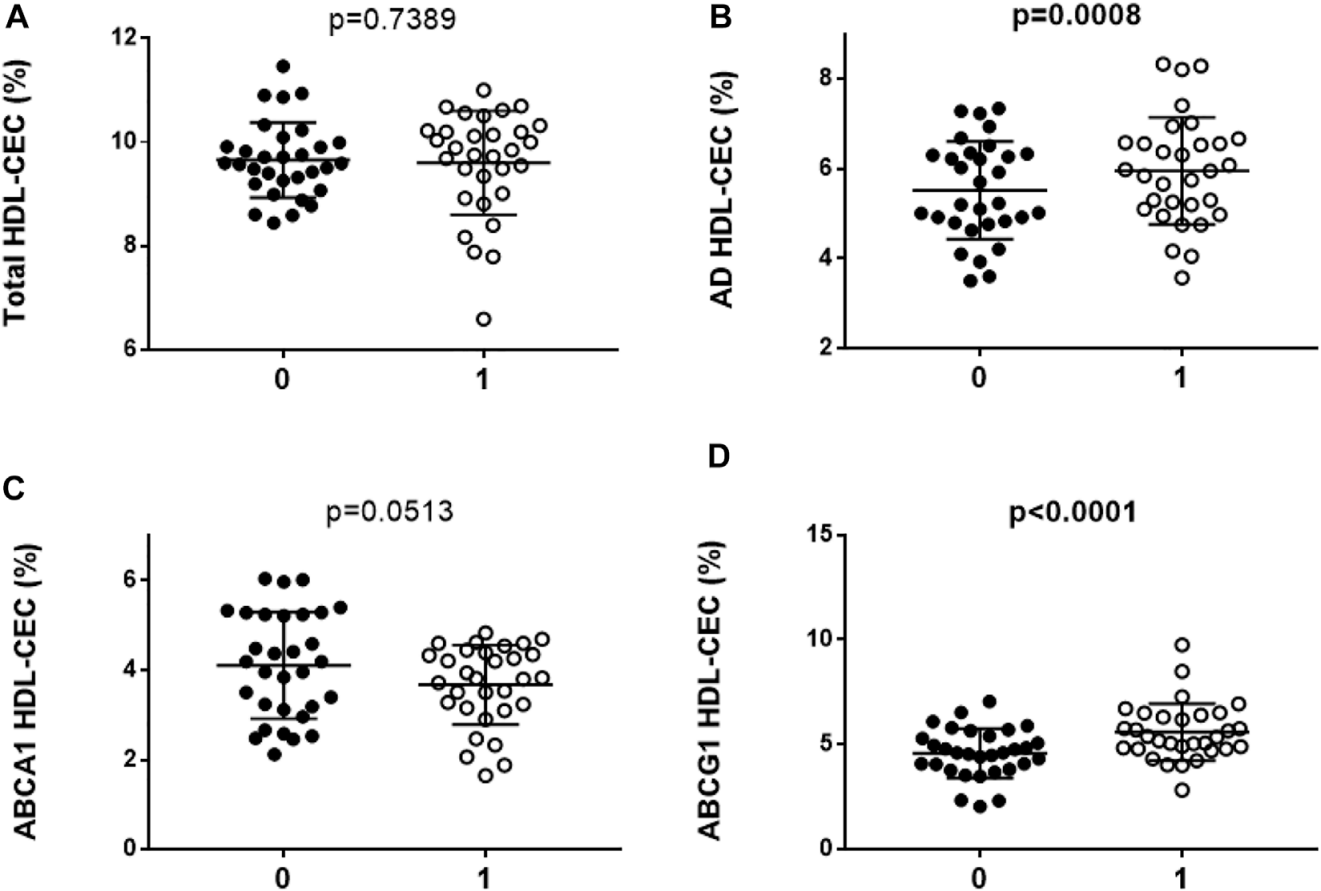
HDL cholesterol efflux capacity (HDL-CEC) in FH subjects (N = 31) before and after PCSK9-i treatment. **(A)**: total HDL-CEC; **(B)**: AD HDL-CEC; **(C)**: ABCA1 mediated HDL-CEC; **(D)**: ABCG1 mediated HDL-CEC. Every dot of the graphs represents the mean percentage of each triplicate analyses for serum sample. The horizontal line is the mean of each group. 0: FH subjects before PCSK9-i treatment; 1: FH subjects after 6 months of PCSK9-i treatment. Values in bold indicate statistically significant results.

On the FH patient cohort, we first evaluated the capacity of serum HDL to promote cholesterol efflux (HDL-CEC) (Figure 1). No significant difference in total HDL-CEC from cAMP-stimulated J774 was observed in FH subjects after 6 months of PCSK9-i (*p* = 0.7389) (Figure 1A). However, by analyzing the two pathways contributing to total HDL-CEC, we found a significant increase of AD HDL-CEC (+7.8%; *p* = 0.0008; Figure 1B); however, PCSK9-i treatment promoted a trend towards a reduction of ABCA1 mediated HDL-CEC, without reaching statistical significance (*p* = 0.0513) (Figure 1C). Treatment with PCSK9-i markedly increased ABCG1 mediated HDL-CEC (+22.2%; *p* < 0.0001; Figure 1D). In two secondary analyses, we stratified FH patients according to smoking and hypertension, respectively. We found that non-smokers as well as non-hypertensive patients exhibited a significant increase of the ABCG1 mediated HDL-CEC after 6 months of PCSK9-i treatment (Supplementary Tables S1, S2, Supplementary Material). Interestingly, by comparing HDL-CEC of treated FH patients with that of a small group of control subjects added in each experiment as internal standards, we observed similar values (AD HDL-CEC of controls: 5.9% ± 0.7, AD HDL-CEC of FH after treatment 6.0% ± 1.2, *p* > 0.999; ABCG1 HDL-CEC of controls: 5.2% ± 1.7, ABCG1 HDL-CEC of FH after treatment 5.6% ± 1.4, *p* > 0.9999; ABCA1 HDL-CEC of controls: 3.6% ± 0.6; ABCA1 of FH after treatment 3.7% ± 0.9, *p* > 0.9999).

### 3.3 Serum cholesterol loading capacity

**FIGURE 2:**
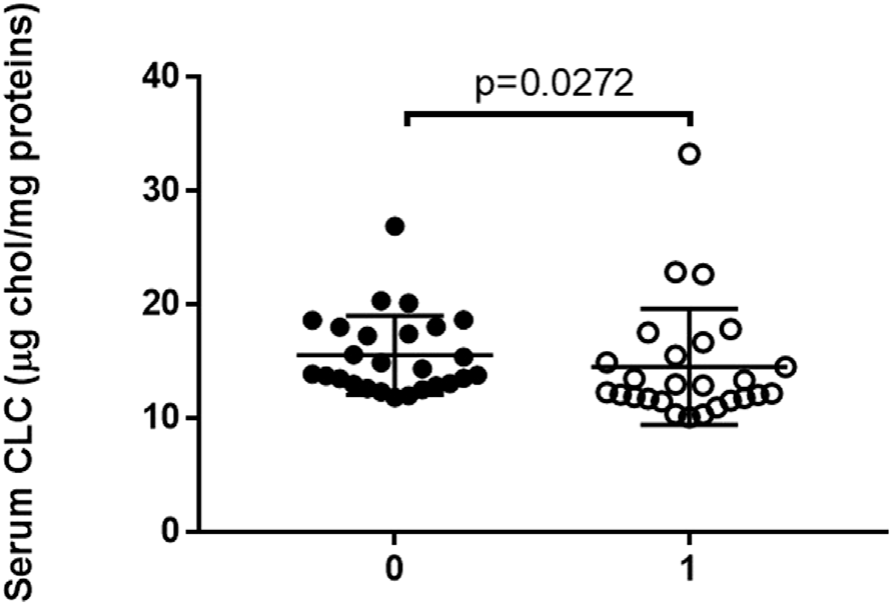
Serum cholesterol loading capacity (CLC) in FH subjects (N = 26) before and after 6 months of PCSK9-i treatment. Every dot of the graph represents the mean percentage of each triplicate analyses for serum sample. The horizontal line is the mean of each group. 0: FH subjects before PCSK9-i treatment; 1: FH subjects after 6 months of PCSK9-i treatment. 5 samples over 31 were not available for CLC determination.

As cell cholesterol content is the result of cholesterol efflux and influx, we evaluated whether PCSK9-i treatment is associated to changes in the pro-atherogenic potential of the serum by measuring its cholesterol loading capacity (CLC) in macrophages. We found that serum CLC was significantly decreased in FH subjects after 6 months of PCSK9-i treatment (−6.6%, *p* = 0.0272; Figure 2). Additionally, we found a direct but weak correlation between serum CLC and LDL-c serum levels (R^2^ = 0.091; *p* = 0.006). In two secondary analyses, after stratification of FH patients according to smoking and hypertension respectively, we observed a significant reduction of serum CLC in smokers and hypertensive patients after 6 months of PCSK9-i treatment (Supplementary Tables S1, S2, Supplementary Material). As for HDL-CEC, CLC values of sera from FH patients after PCSk9-i treatment were similar to those of a small group of control sera added in each experiment as internal standards (CLC of control sera: 11.6 ± 1.7; CLC of FH after treatment: 14.5 ± 5.1; *p* = 0.4158).

## 4 Discussion

In the era of novel circulating cardiovascular biomarkers, an emerging interest has been observed for the potential role of HDL cholesterol efflux capacity (CEC) in the pathogenesis and progression of ASCVD (Lee et al., 2021). This open-label, observational study focused on the effect of PCSK9 inhibitors (PCSK9-i) on plasma lipoprotein functions, i.e., HDL-CEC and serum cholesterol loading capacity (CLC) in FH subjects. CEC indicates the HDL antiatherogenic activity independent of its plasma level (Rohatgi et al., 2021). We found that in FH patients PCSK9-i therapy significantly enhanced the ABCG1 and the aqueous diffusion-mediated HDL-CEC, while it significantly reduced serum CLC, a marker of serum proatherogenic potential, linked to an enhanced CV risk (Adorni et al., 2019b). According to our knowledge, this is the first study evaluating PCSK9-i effect on the functional lipid profile, namely the serum lipoprotein functions, in a cohort of FH subjects. These effects could be of great clinical relevance in these subjects, since an impaired HDL-CEC (Nenseter et al., 2013) and an increased serum CLC were previously documented (Adorni et al., 2012). Indeed, the comparison between CEC and CLC values of FH patients after PCSK9-i treatment and those of a small group of control sera added in our experiments as internal standards suggests that the treatment seems able to retrieve HDL-CEC and serum CLC to normal values. Thus, the beneficial effect of PCSK9-i on the functional lipid profile could contribute to restore the normal lipoprotein functions and eventually to the CV benefit of these drugs beyond LDL cholesterol (LDL-c) reduction in high CV risk subjects such as FH.

As concerns the blood lipid profile, the directional changes observed for total cholesterol and LDL-c after treatment with PCSK9-i were in line with previous reports of Ge et al. who described similar results from PCSK9-i clinical trials involving FH subjects (Ge et al., 2021). These results were obtained independently of smoking or hypertension. However, differently to what reported by Ge and colleagues (Ge et al., 2021), in which PCSK9-i led to a slight rise in HDL-c and to a reduction of triglycerides, in our cohort, treatment did not significantly change HDL-c and triglyceride levels, even though the latter tent to decrease. This discrepancy may be possibly attributable to the relatively small sample size in the current study or to specific intrinsic characteristics of our subjects.

In our study the observed increased ABCG1 HDL-CEC after PCSK9-i treatment occurred despite no changes in HDL-c levels, suggesting that HDL-CEC increase may reflect a relative increase amount and/or efficiency of mature HDLs that are the main cholesterol acceptors for this efflux pathway (Sankaranarayanan et al., 2009). The important improvement in HDL function through ABCG1 observed in this study may thus suggest that a HDL remodeling towards mature particles (Asztalos et al., 2017) may occur after PCSk9-i treatment. In line with our findings, it has been previously reported that PCSK9-i treatment is associated with an increase of medium-sized HDL particles (Ingueneau et al., 2020), responsible for CEC mainly through ABCG1 (Sankaranarayanan et al., 2009).

Only two studies investigated the effect of PCSK9-i on HDL functions. Lappegård et al. (2018) firstly explored HDL-CEC on lipoprotein apheresis and subsequently on PCSK9 inhibition with evolocumab in FH subjects, finding no changes in HDL-CEC during PCSK9-i treatment. However, the sample size in this study was particularly small (three subjects) and the *ex vivo* CEC evaluation was performed in a different cellular model and with a different methodological assay, thus implying the need of a careful interpretation of their results.

More recently, Ying et al. (2022) analyzed the effects of evolocumab and atorvastatin on CEC in healthy normolipidemic subjects and they found that evolocumab decreased CEC of whole plasma, thus considering the mixture of all lipoproteins present in serum. As the authors suggested, this effect was likely related to the decrease in apoB-containing lipoproteins induced by the treatment. Looking at the effects of the drug on the isolated serum HDL fraction capacity to promote ABCA1 and AD-mediated HDL-CEC, no significant influence of the treatment was observed by the authors on both the efflux pathways. In this regard, our data are consistent with the lack of a significant effect of PCSK9-i treatment on ABCA1 HDL-CEC, however, different conclusions came from our study with respect to HDL-CEC through AD processes that was improved together with ABCG1 HDL-CEC by PCSK9-i treatment. Compared to the study by Ying, our findings showed an increase of HDL-CEC after PCSK9-i treatment in a clearly different experimental setting, i.e., healthy normolipidemic subjects vs. FH subjects characterized by a higher atherosclerotic burden and higher CV risk. This likely implies a different impact of the treatment on the lipoprotein functional profile and a different therapeutic strategy impact in clinical practice.

In addition to the role in cholesterol homeostasis, ABCG1 HDL-CEC could also affect inflammatory signaling in macrophages (Prosser et al., 2012). This link is supported by the results of clinical studies in which a specific association between ABCG1 HDL-CEC impairment and inflammation indexes has been demonstrated (Ronda et al., 2014; Zimetti et al., 2017). Taking into consideration these findings, the improvement of ABCG1-efflux induced by PCSK9-i treatment could ameliorate the inflammatory status that characterized subjects with a high cholesterol burden, such as FH (Bahrami et al., 2020). In line with this consideration, a recent study by in a cohort of FH subjects, found that PCSK9-i ameliorated systemic inflammation by promoting the activation of T-regulatory cells Marques et al. (2022). Thus, PCSK9 could be considered also an intriguing new player of the immune-inflammation axis and its inhibition could be a primary target in a chronic inflammatory disease such as atherosclerotic processes, beyond PCSK9-i related LDL-c reduction.

As macrophage cholesterol storage is the result of efflux-influx mechanisms, it was previously shown that the serum CLC analysis is needed to assess the positive effect of treatment on overall serum atherogenic properties in foam cell pathogenesis (Greco et al., 2020). In line with this concept, in our study for the first time we observed decrease of CLC in FH patients treated with PCSK9-i treatment indicates that the likely PCSK9-i related arterial wall foam-cell formation reduction could contribute to the proven CV benefit associated with this treatment (Steffens et al., 2020).

Despite a reduction in LDL-c levels observed after PCSK9-i treatment, only a weak correlation between CLC and LDL-c levels was observed, suggesting a specific effect of treatment on the overall ability of serum to deliver cholesterol to cells (CLC). In fact, previous studies documented how the accumulation of cholesterol is not always dependent on the circulating levels of LDL-c but can correlate with the quality and/or function of these lipoproteins (van Velzen et al., 2021). Among LDL subclasses, oxidized LDL (oxLDL) have an increased ability to induce macrophage cholesterol accumulation (Yu et al., 2013); of note, it was previously shown that evolocumab significantly reduced the content of circulating oxLDL and small LDL in particular, in patients with coronary artery disease (Lankin et al., 2018) (Li et al., 2021). Moreover, PCSK9-i treatment reduced lipoprotein (a) [Lp (a)] plasma levels (Kasichayanula et al., 2018; O’Donoghue et al., 2019), an atherogenic lipoprotein highly prone to oxidation and penetration through endothelia promoting the foam cell formation (Ugovšek and Šebeštjen, 2021). Thus, it is likely that the PCSK9-i could impact on circulating apoB-rich lipoproteins not only quantitatively but also qualitatively improving the overall lipid profile, and possibly explaining the lower CLC observed in our cohort of FH subjects.

The present study has some limitations: first, this was not a randomized study and the PCSK9-i therapeutic strategy is subsequent to a physician’s choice. Furthermore, the sample size was relatively small, even though it was enough to demonstrate HDL-CEC and serum CLC changes after PCSK9-i treatment in a real-life setting. This small sample size very likely determined some loss of statical significance after stratification of HDL-CEC and serum CLC between smokers and non-smokers, as well according to the absence or presence of hypertension, so that wider studies will be necessary to establish the influence of these two factors.

This preliminary report should be validated in a wider FH cohort by providing additional mechanistic insights to our findings, by evaluating, for example, HDL metabolism as well as changes of HDL or LDL size, HDL remodeling enzyme activity and HDL/LDL lipid or protein composition that would explain the changes in serum lipoprotein functions that we observed. However, it is worth mentioning that the current study is the first involving FH patients to explore the effect of PCSK9-i on the main HDL-CEC pathways and on serum CLC in clinical practice. An additional limitation of the present work may also be related to the unavailability of a CV risk endpoint. However, it was previously found that PCSK9-i treatment ameliorated the pulse wave velocity (PWV), a biomarker of early cardiovascular injury, in a cohort of LDL off-target FH subjects (Scicali et al., 2021).

In conclusion, PCSK9-i treatment significantly increased ABCG1-mediated HDL-CEC as well as CEC by the aqueous diffusion pathway and it reduced serum CLC in a cohort of FH subjects. Our findings clearly showed that the favorable effect of PCSK9-i treatment on lipoprotein functions could contribute to the CV benefit of these drugs beyond LDL-c reduction in subjects at high CV risk such as FH in clinical practice; however, a designed study with a wider cohort of subjects is needed to assess the effect of PCSK9-i therapy on these pathways in FH subjects.

## Data Availability

The raw data supporting the conclusion of this article will be made available by the authors, without undue reservation.
