# Supplementary material for: Effects of PCSK9 inhibitors on HDL cholesterol efflux and serum cholesterol loading capacity in familial hypercholesterolemia subjects: a multi-lipid-center real-world evaluation
Source: Front Mol Biosci. 2022 Jul 19;9:925587. doi: 10.3389/fmolb.2022.925587 (PMC9343790; doi:10.3389/fmolb.2022.925587)
Supplement: Supplementary file 1 [file DataSheet1.docx]

Table 1S. HDL CEC, serum CLC and lipid profile in smokers (A) and non-smokers (B) at baseline and after six months of PCSK9-i treatment

A

| **SMOKERS** | | | **p-value** |
| --- | --- | --- | --- |
|  | **Before treatment**  (n=25) | **After 6-month PCSK9-i treatment**  (n=25) |  |
| Total HDL-CEC (%) | 9.69 ± 0.72 | 9.49 ± 1.07 | 0.439 |
| AD HDL-CEC (%) | 5.44 ± 1.05 | 5.89 ± 1.27 | 0.182 |
| ABCA1 HDL-CEC (%) | 4.37 (3.15-5.28) | 3.80 (3.20-4.37) | 0.102 |
| ABCG1 HDL-CEC (%) | 4.61 (4.08-5.52) | 5.39 (4.91-6.45) | **0.007** |
| Serum CLC (µg/mg protein)* | 13.74 (12.88-15.04) | 15.25 (12.98-28.84) | **0.007** |
| TC (mg/dL) | 238 (216-287) | 139 (106-182) | **<0.0001** |
| LDL-c (mg/dL) | 139 (105-171) | 61 (40-110) | **<0.0001** |
| HDL-c (mg/dL) | 55 ± 10 | 53 ± 9 | 0.543 |
| TG (mg/dL) | 112 (74-128) | 95 (73-116) | 0.277 |

B

| **NON-SMOKERS** | | | **p-value** |
| --- | --- | --- | --- |
|  | **Before treatment**  (n=6) | **After 6-month PCSK9-i treatment**  (n=6) |  |
| Total HDL-CEC (%) | 9.49 (8.81-10.05) | 10.13 (9.73-10.31) | 0.132 |
| AD HDL-CEC (%) | 5.7 (4.74-7.24) | 6.14 (5.29-7.12) | 0.485 |
| ABCA1 HDL-CEC (%) | 4.07 (2.43-4.26) | 4.02 (3.05-4.54) | 0.563 |
| ABCG1 HDL-CEC (%) | 3.94 (2.27-5.02) | 4.75 (3.88-6.27) | 0.180 |
| Serum CLC (µg/mg protein)* | 13.74 (12.88-15.04) | 15.25 (12.98-28.84) | 0.486 |
| TC (mg/dL) | 237 (191-285) | 125 (112-161) | **0.009** |
| LDL-c (mg/dL) | 164 (127-202) | 54 (39-88) | **0.004** |
| HDL-c (mg/dL) | 46 (41-55) | 52 (45-56) | 0.368 |
| TG (mg/dL) | 109 (67-163) | 93 (58-159) | 0.699 |

*ABCA1: ATP-binding cassette transporter A1; ABCG1: ATP-binding cassette transporter G1; AD: aqueous diffusion; CEC: cholesterol efflux capacity; CLC: cholesterol loading capacity; HDL-c: High Density Lipoproteins; LDL-c: Low Density Lipoproteins; n.s: not significant; PCSK9-i: proprotein convertase subtilisin/kexin type 9 inhibitors; TC: total cholesterol; TG: triglyceride. Data are presented as mean ± SD or median and interquartile range (25th to 75th percentile), according to normal or skewed distribution respectively. Values in bold indicate statistically significant results. *Serum CLC was available for 4/6 samples in the smokers’ group and 22/25 samples in the non-smoker group.*

Table 2S. HDL CEC, serum CLC and lipid profile in subjects with (A) and without hypertension (B) at baseline and after six months of PCSK9-i treatment

A

| **HYPERTENSIVE** | | | **p-value** |
| --- | --- | --- | --- |
|  | **Before treatment**  (n=14) | **After 6-month PCSK9-i treatment**  (n=14) |  |
| Total HDL-CEC (%) | 9.71 ± 0.78 | 9.54 ± 0.94 | 0.569 |
| AD HDL-CEC (%) | 5.09 ± 0.6 | 5.69 ± 1.24 | 0.184 |
| ABCA1 HDL-CEC (%) | 4.59 ± 1.15 | 3.85 ± 0.81 | 0.060 |
| ABCG1 HDL-CEC (%) | 5.01 (3.91-5.82) | 5.67 (4.87-6.51) | 0.140 |
| Serum CLC (µg/mg protein)* | 14.39 (12.96-18.05) | 12.08 (11.54-14.46) | **0.023** |
| TC (mg/dL) | 223 (198-252) | 123 (106-137) | **<0.0001** |
| LDL-c (mg/dL) | 130 (86-163) | 51 (31-63) | **<0.0001** |
| HDL-c (mg/dL) | 51 ± 8 | 53 ± 9 | 0.726 |
| TG (mg/dL) | 112 (72-129) | 96 (82-116) | 0.533 |

| **NON-HYPERTENSIVE** | | | **p-value** |
| --- | --- | --- | --- |
|  | **Before treatment**  (n=17) | **After 6-month PCSK9-i treatment**  (n=17) |  |
| Total HDL-CEC (%) | 9.48 (9.03-10.07) | 9.89 (9.49-10.27) | 0.286 |
| AD HDL-CEC (%) | 5.87 ± 1.01 | 6.16 ± 1.15 | 0.429 |
| ABCA1 HDL-CEC (%) | 3.71 ± 1.09 | 3.52 ± 0.94 | 0.608 |
| ABCG1 HDL-CEC (%) | 4.45 ± 1.05 | 5.40 ± 1.29 | **0.024** |
| Serum CLC (µg/mg protein)* | 15.35 ± 3.02 | 14.87 ± 4.14 | 0.738 |
| TC (mg/dL) | 259 (225-299) | 157 (126-195) | **<0.0001** |
| LDL-c (mg/dL) | 145 (128-199) | 81 (46-116) | **<0.0001** |
| HDL-c (mg/dL) | 55 ± 13 | 53 ± 9 | 0.491 |
| TG (mg/dL) | 99 (72-135) | 92 (61-122) | 0.293 |

B

*ABCA1: ATP-binding cassette transporter A1; ABCG1: ATP-binding cassette transporter G1; AD: aqueous diffusion; CEC: cholesterol efflux capacity; CLC: cholesterol loading capacity; HDL-c: High Density Lipoproteins; LDL-c: Low Density Lipoproteins; n.s: not significant; PCSK9-i: proprotein convertase subtilisin/kexin type 9 inhibitors; TC: total cholesterol; TG: triglyceride. Data are presented as mean ± SD or median and interquartile range (25th to 75th percentile), according to normal or skewed distribution respectively. Values in bold indicate statistically significant results. *Serum CLC was available for 13/14 samples in the hypertensive group and 13/17 samples in the non-hypertensive group.*
